# Higher Order Organization of the mtDNA: Beyond Mitochondrial Transcription Factor A

**DOI:** 10.3389/fgene.2019.01285

**Published:** 2019-12-20

**Authors:** Dan Mishmar, Rotem Levin, Mansur M. Naeem, Neal Sondheimer

**Affiliations:** ^1^ Department of Life Sciences, Ben-Gurion University of the Negev, Beer-Sheva, Israel; ^2^ Institute of Medical Sciences and the Department of Paediatrics, The University of Toronto, Toronto, ON, Canada

**Keywords:** ATAC-seq, DNase-seq, G-quadruplex, higher order organization, mtDNA, mitochondrial transcription factor A

## Abstract

The higher order organization of eukaryotic and prokaryotic genomes is pivotal in the regulation of gene expression. Specifically, chromatin accessibility in eukaryotes and nucleoid accessibility in bacteria are regulated by a cohort of proteins to alter gene expression in response to diverse physiological conditions. By contrast, prior studies have suggested that the mitochondrial genome (mtDNA) is coated solely by mitochondrial transcription factor A (TFAM), whose increased cellular concentration was proposed to be the major determinant of mtDNA packaging in the mitochondrial nucleoid. Nevertheless, recent analysis of DNase-seq and ATAC-seq experiments from multiple human and mouse samples suggest gradual increase in mtDNA occupancy during the course of embryonic development to generate a conserved footprinting pattern which correlate with sites that have low TFAM occupancy *in vivo* (ChIP-seq) and tend to adopt G-quadruplex structures. These findings, along with recent identification of mtDNA binding by known modulators of chromatin accessibility such as MOF, suggest that mtDNA higher order organization is generated by cross talk with the nuclear regulatory system, may have a role in mtDNA regulation, and is more complex than once thought.

## Introduction

The genome of all organisms undergoes concerted cycles of packaging to reduce its volume and to control access to the regulatory mechanisms of transcription and replication. In the eukaryotic nucleus, DNA is compacted into chromatin, which provides differential accessibility in response to a variety of histone modifications (Zhu and Li, 2016). The bacterial genomes, which lack histones, are folded into nucleoids using a set of dedicated proteins, entitled Nucleoid-Associated Proteins (NAPs), such as HU, Histone-like Nucleoid Structuring protein (H-NS) and Structural Maintenance of Chromosomes proteins (SMC). Alongside their architectural role in DNA packaging, these proteins also play a role in other processes, such as replication and chromosome segregation (Badrinarayanan et al., 2015; Dame and Tark-Dame, 2016). Notably, the most commonly used models for investigation of nucleoid organization are *Escherichia coli*, *Bacillus subtilis* and *Caulobacter crescentus* (Dame and Tark-Dame, 2016); the latter is an alphaproteobacterium, which belongs to the same phylogenetic branch from which the mitochondria originated (Wang and Wu, 2015).

The circular genomes of *C. crescentus* are organized in ellipsoidal and helical structures between two opposite poles of the cell, creating two ‘arms’ that are folded around each other (Le et al., 2013). While analyzing interactions between different regions within the *C. crescentus* genome by genome-wide chromatin conformational capture (Hi-C) (Le et al., 2013), 23 preferential Chromosomal Interaction Domains (CID) were identified. CID boundaries seem to closely associate with transcription and replication units. The boundaries tend to reestablish shortly after, or even during DNA replication, possibly to disentangle the newly formed DNA molecules. Additionally, the CID boundaries can be disrupted by transcription inhibition (Le et al., 2013). Novel CID boundaries can be created by artificially moving loci of highly expressed genes into inherently low expressed regions (Le et al., 2013). These findings, strongly suggest that the bacterial nucleoid, including that of alphaproteobacteria, is a highly regulated structure with great importance to DNA replication and transcription.

In addition to their nuclear genome, all eukaryotic cells contain a much smaller cytoplasmic genome—the mitochondrial DNA (mtDNA). This genome originated ~2.5 billion years ago from an ancient endosymbiosis between a former free-living alphaproteobacterium and the progenitor of all eukaryotic cells (Sagan, 1967; Pittis and Gabaldon, 2016). Although during the course of evolution the ancient bacterium lost most of its inherent genetic material either due to transfer to the nucleus, or due to natural selection, the mitochondria in the vast majority of eukaryotes still harbor their own genomes. Despite its modest size, the mammalian mtDNA encodes 13 critical subunits of the oxidative phosphorylation system (OXPHOS), two ribosomal RNA genes and 22 tRNAs that are required for cellular energy production. Mammalian mtDNA forms a protein-DNA structure that was termed ‘nucleoid’, to highlight its ancient bacterial heritage (see below). The animal mtDNA is four orders of magnitude smaller than the nuclear genome, and has been long thought to be separately regulated from the nuclear genome (Gustafsson et al., 2016). Accordingly, mitochondrial transcription factor A (TFAM) is believed to be sufficient for mitochondrial nucleoid formation (Kaufman et al., 2007) and the primary driver of mtDNA packaging (Gustafsson et al., 2016; Farge and Falkenberg, 2019). The role of TFAM in mtDNA packaging and higher order organization has been recently thoroughly reviewed, and therefore will be mentioned here only briefly (Farge and Falkenberg, 2019). TFAM is highly conserved across species, and despite the apparently linear mtDNA organization in yeast (Gerhold et al., 2010), the yeast orthologue (Abf2p) of TFAM packs this genome as well (Farge and Falkenberg, 2019). mtDNA condensation positively correlates with the cellular concentration of TFAM so that increased TFAM concentration leads to higher degrees of mtDNA compaction (Kukat et al., 2015).

Thus, our current view of mtDNA regulation suggests that a nuclear-encoded yet mitochondrially restricted set of proteins modulates mtDNA transcription, replication and packaging (Gustafsson et al., 2016). For example, mtDNA genes are transcribed by POLRMT, and not RNA Polymerase II which transcribes nuclear mRNAs, and the mtDNA is replicated by DNA polymerase gamma (POLG), which has no accepted role in replication of the nuclear DNA. However, it would be surprising from an evolutionary point of view if the past 2.5 billion years since mitochondrial endosymbiosis had not led to significant adaptation of the regulation of the mitochondrial and nuclear DNA. Is it plausible that the longtime co-existence of the mitochondrion and its host have been accompanied by adaptation of mtDNA to the host regulatory and packaging systems? Co-adaptation of the nuclear and mitochondrial genomes had been demonstrated in the context of the OXPHOS and in the mitoribosomes, which use nuclear DNA-encoded proteins, and either exclusively mtDNA-encoded proteins (in OXPHOS) or mtDNA-encoded rRNA and tRNA transcripts (in the mitoribosome) (Levin et al., 2014). However, the discovery of transcription factors that directly regulate transcription in both the nucleus and in the mtDNA has suggested that the control of gene expression is coordinated not only by signals, but by dual localization of transcription factors (Barshad et al., 2018). Hence, adaptation of mtDNA regulation to the nuclear regulatory system is plausible.

Mitochondrial DNA is compacted through its interactions with TFAM (Kukat et al., 2015), but there is growing evidence for the involvement of additional nuclear-encoded proteins that also regulate nuclear chromatin. This includes MOF (Chatterjee et al., 2016), members of the AP1 family (c-Jun and JunD) as well as CEBPB (Blumberg et al., 2014) and MEF2D (She et al., 2011). The discovery of mtDNA binding and mitochondrial transcriptional regulation by MOF, a histone lysine acetyltransferase that remodels chromatin, was particularly surprising, as it raises questions about its acetyltransferase target in the mitochondria, and its possible role in mtDNA organization. Secondly, c-Jun and JunD, which were recently shown to bind negatively selected sites in the mtDNA (Blumberg et al., 2014), tend to bind nuclear DNA enhancer regions and affect nuclear DNA gene regulation (Phanstiel et al., 2017). Third, CEBPB, a known chromatin remodeler (Bornstein et al., 2014), not only binds the mtDNA *in vivo*, but also serves as a candidate repressor of human mtDNA gene expression (Barshad et al., 2018). Fourth, DNase-seq and ATAC-seq analysis in multiple human and mouse cells revealed a conserved footprinting pattern, which overlapped known mtDNA regulatory elements, yet correlated with low TFAM occupancy in HeLa cells (Blumberg et al., 2018). This ATAC-seq mtDNA footprinting pattern was gradually formed during the course of embryogenesis in both mouse and humans, as reflected by gradually increasing mtDNA occupancy (Marom et al., 2019). Hence, it is possible that there are mtDNA sites which are consistently occupied, and sites that are consistently under-occupied across the mtDNA, and that the mtDNA is bound not only by TFAM but rather by other additional proteins in an organized manner. This reflects the existence of an organized protein–DNA structure in the mitochondrial genome, thus providing first clues for the existence of a structured higher order organization of the mitochondrial genome.

**Figure 1:**
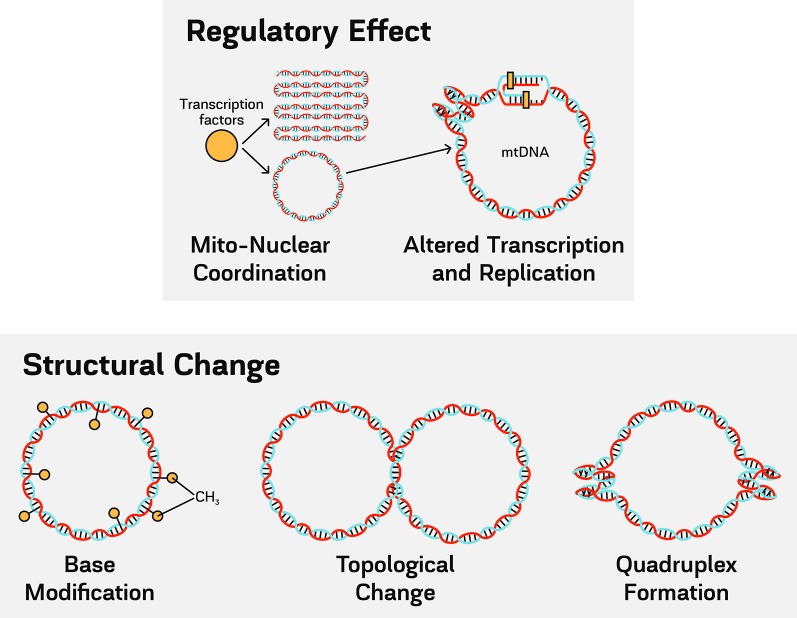
The possible structural changes in the mtDNA and the cross-talk between mtDNA higher order organization and regulation.

We would argue that the investigation of the regulation of the mitochondrial nucleoid in the frame of protein-DNA patterns of interactions and their impact on regulation of mtDNA gene expression and replication is of equivalent importance to our understanding of the organization and compaction of the nuclear chromosome, but that it is markedly less well studied and understood. In this essay we will discuss current knowledge regarding the nature of the higher order organization of the mitochondrial genome (**Figure 1**), and assess its functional potential from an evolutionary perspective.

### Mitochondrial Nucleoid and mtDNA Content

The only known structural unit of the mitochondrial genome is the nucleoid, which contains mtDNA and closely interacting proteins (Hensen et al., 2014). The nucleoids are vital for mitochondrial function as they coordinate transcription (Rebelo et al., 2011), translation (He et al., 2012) and interact with enzymatic activities of the mitochondrial inner membrane (Wang and Bogenhagen, 2006). There are a correspondingly large number of proteins that can be pulled down by crosslinking to nucleoids, thus reflecting these diverse activities (Bogenhagen et al., 2008). High-resolution microscopy techniques were used to show that nucleoids are compact and ellipsoidal, suggesting the exclusion of non-nucleoid proteins, and that nucleoids are associated with the mitochondrial inner membrane (Brown et al., 2011; Kukat et al., 2011; Kukat et al., 2015). The number of mtDNA molecules in each nucleoid has been a matter of considerable debate. Logically, the nucleoids must at least transiently contain multiple mitochondrial genomes after the completion of replication. Most studies have observed that the copy number is stably higher with estimates ranging from 1.4 to 7.5 genomes per nucleoid [(reviewed in (Lee and Han, 2017)].

### MtDNA Folding and Loops—Current and Future Studies

Higher order organization of both the eukaryotic nuclear genome and the bacterial nucleoid involve regulated steps of protein binding followed by bending and folding to allow the interaction of sequences that are distant in the primary DNA sequence. In a study of the mtDNA binding pattern of the mitochondrial transcription termination factor MTERF1 in mammalian cells, simultaneous binding of MTERF1 was observed at the proximal heavy strand promoter (HSP1) and within the MT-TL1 sequence (Martin et al., 2005). This interaction increased the expression of genes regulated by HSP1, and a model was proposed that would allow the direct recycling of transcription complexes from the termination point of HSP1 transcription back to its origin. While attractive, the phenotype of an Mterf1 deficient mouse cast doubt upon this elegant concept, as the predicted loss in HSP1 activity was not observed (Terzioglu et al., 2013). Other *cis* interactions along mtDNA are yet to be discovered. Interactions between distant nuclear genomic regions are currently being investigated using sequencing-based techniques such as the Chromosome conformation capture (3C) and subsequent derivative of this methodology (i.e., 4C, 5C and HiC) (Oluwadare et al., 2019), yet all of these techniques are currently designed to identify interactions between regions that are megabases apart, which limit their utility in the study of the human mtDNA. Although a recent study of HiC data claimed to observe direct interactions between the mitochondrial and nuclear genomes (Doynova et al., 2016), no independent study supported such findings. Given the above, there is a need for the development of techniques that will allow mapping of interactions between mtDNA sequences, while taking into account the small size of this genome and its circularity.

### Mitochondrial Transcription Responds to Structural Cues Along mtDNA

Mitochondrial transcription uses unique features to allow the differential expression of a very tightly packed genome with a limited number of primary transcripts. Since mitochondrial transcription has recently been reviewed (Gustafsson et al., 2016), here we will only consider the role of the physical structure of mtDNA in the initiation and termination of transcription.

One key challenge for mitochondrial transcription is the use of oppositely oriented promoters that transcribe the same regions in both directions—with strand specific promoters in mammals but bidirectional promoters in birds (L’Abbe et al., 1991; Randi and Lucchini, 1998) and amphibians (Bogenhagen et al., 1986; Bogenhagen and Romanelli, 1988). The human mtDNA harbors a single light-stranded promoter, which is responsible for expression of the OXPHOS complex I subunit *ND6* as well as eight tRNA. The activation of this promoter requires the binding of TFAM, which creates a pronounced U-turn bending in mtDNA, proximal to the site of transcription initiation (Ngo et al., 2011; Rubio-Cosials et al., 2011). The two heavy-strand promoters are closely adjacent to each other, with HSP1 principally driving expression of the two rRNA genes (i.e. the 12S and 16S rRNAs), and HSP2 driving the expression of the remaining twelve protein-coding genes and the distal tRNA genes along the heavy strand (Montoya et al., 1983). Like LSP, TFAM activates HSP1, although studies have come to different conclusions as to the topology of TFAM’s interaction at HSP1 (Ngo et al., 2014; Morozov and Temiakov, 2016; Hillen et al., 2017; Uchida et al., 2017).

The balancing of expression of HSP1 and HSP2 has been a matter of some debate. It seems reasonable that some mechanism must exist, since HSP1 is primarily devoted to rRNA and HSP2 to the expression of protein coding genes, which was also shown in living cells (Blumberg et al., 2017). Our group and others have shown that HSP2 is distinct in that TFAM is not only dispensable for activation, but actively inhibits it (Lodeiro et al., 2012; Zollo et al., 2012). We have further shown that the topological state of mtDNA may be important for HSP2 activation. Unique among the promoters, HSP2 is activated by negative supercoiling in a fashion reminiscent of bacterial systems, but no similar effect is seen at LSP and HSP1 (Zollo and Sondheimer, 2017).

The termination of mitochondrial transcription is also regulated by the physical state of mtDNA. Because the molecule is circular and has oppositely oriented promoters, processive transcriptional complexes are at risk of collision. Because of the positioning of genes, the termination of LSP and HSP1 at a point between mt.3229 (the end of the 16S ribosomal RNA as transcribed by HSP1) and mt.4329 (the end of MT-TQ as transcribed by LSP) would allow the simultaneous utilization of LSP and HSP1 without promoter collision. Considerable evidence has been provided for the role of MTERF1 in interaction with mt.3232–3253 (within the coding sequence of MT-TL1), including the crystal structure of the interaction of MTERF1 with its mtDNA target sequences (Yakubovskaya et al., 2010). As noted above, evidence from mouse knockout studies of *Mterf1* agreed only partially with this concept, and suggested that the insulation of the LSP against transcription proceeding back through the promoter might also be important (Terzioglu et al., 2013). It is important to recognize that the regulation of both transcription and mtDNA physical structure in mouse and human may not be identical, but the organization of mtDNA regulatory elements clearly influences interactions with transcription factors to exert control over gene expression. The means of controlling interactions between transcriptional complexes arising from LSP and HSP2 remains undiscovered.

### Could mtDNA Packaging and Regulation Be Affected by G-Quadruplex (GQ) Formation?

G-Quadruplexes (GQs) are non-canonical nucleic acid secondary structures that use Hoogsteen hydrogen bonding between guanines on the same strand (Rhodes and Lipps, 2015). The occurrence of GQs within the DNA is not random, and is notably conserved across species, thus supporting selective constraints and hence potential functional importance (Murat and Balasubramanian, 2014). Moreover, whereas transient GQs correlate with binding sites of chromatin remodeling-related transcription factors, genome-wide sites with more stable GQs have been implicated in replication stalling and inhibition of chromatin remodeling (Varizhuk et al., 2019), which support their involvement in regulation of higher order DNA organization. For example, GQ-ChIP-seq experiments revealed that most GQs tend to form within nucleosome-depleted regions with increased transcription activity (Hansel-Hertsch et al., 2016). As GQ structures are mostly resolved by RecQ helicases (Mendoza et al., 2016; Sauer and Paeschke, 2017; Varizhuk et al., 2019), it is noteworthy that one such helicase, RecQ4, is transported into the mitochondria, interacts with DNA POLG and promotes mtDNA replication (Ding and Liu, 2015). Indeed, due to the asymmetric composition of nucleotides in the heavy (more guanine-rich) and light (more cytosine-rich) strands of mtDNA, the heavy mtDNA strand is prone to GQ formation. Previously, *in silico* analysis suggested the existence of G-quadruplex-forming motifs throughout the human mtDNA (Falabella et al., 2019). Imaging of mtDNA using GQ binding dyes showed that they are widely present (Huang et al., 2015), and the application of compounds that bind to GQ impact mtDNA transcription and replication (Falabella et al., 2019). We have recently demonstrated that GQ formation can even selectively bias the replication of a mixed mtDNA population (heteroplasmy) (Naeem et al., 2019). Hence, similar to the nuclear genome, GQ formation in the human mtDNA affects the regulation of this genome.

Although *in vitro* experiments suggested that TFAM binds to GQ at non-physiological concentrations (Lyonnais et al., 2017), analysis of ChIP-seq TFAM binding experiments in HeLa cells revealed TFAM occupancy throughout the mtDNA (Wang et al., 2013), yet low occupancy of TFAM at GQ-forming regions (Blumberg et al., 2018). Moreover, we showed that G-quadruplex-forming motifs tend to co-localize with conserved DNase-seq footprinting sites in adult cells (Blumberg et al., 2018) and during development (Marom et al., 2019). Other proteins such as the ATP-dependent Lon protease bind GQ sequences *in vitro* (Lu et al., 2003), and *in vivo* (Lu et al., 2007). Thus, it is plausible that investigation of the conformation assumed by such motifs *in vivo* will offer clues for the discovery of novel mtDNA binding proteins that may be involved in the construction and regulation of its higher order organization. Interestingly, nuclear DNA regions that tend to be packed late during the cell cycle, and are prone to breakage, also harbor non-B DNA structures (Dong et al., 2014). Specifically, GQ structures are resolved at the DNA, likely by the Pif1 helicase, to allow maintenance of the mtDNA (Bannwarth et al., 2016). Indeed, double mutant *Pif1* mice exhibit elevated levels of mtDNA damage. As in the nuclear genome, hotspots for chromosomal aberrations and fragile sites tend to correlate with the state of chromatin accessibility (Mishmar et al., 1999). Further investigating the patterns of non-canonical DNA structure may offer additional insights to differential accessibility of sites across the mitochondrial genome.

### Structural mtDNA Aberrations in Aging and Disease: Potential Impact on the Higher Order mtDNA Organization

Chromosomal aberrations of various types in the nuclear genome (i.e. inversions, deletions, insertions, duplications and translocations) not only change the location of genes, but also change the location of regulatory elements, thus changing the chromatin structure and regulatory landscape of the modified region. As discussed above, regulatory factors bind the mtDNA not only within the non-coding promoters’ region, but rather throughout the mitochondrial genome [reviewed in: (Barshad et al., 2018)].

Therefore, it is logical that mtDNA aberrations such as deletions, duplications, inversions and insertions may not only change the coding content, but will change the location of regulatory elements and hence have the potential impact on mtDNA regulation. Consistent with this hypothesis, and because of the high gene density of mtDNA, structural rearrangements and deletions are poorly tolerated. The association between mitochondrial deletions and pathology is robust. The accumulation of deletions during the process of aging was discovered nearly thirty years ago (Cortopassi and Arnheim, 1990) and at nearly the same time it was recognized that the Kearns-Sayre syndrome was also linked to deletions in mtDNA (Shoffner et al., 1989). The phenotypic impact of mtDNA deletions has been largely interpreted as the result of the loss of genetic material. The effect of such mtDNA aberrations on mtDNA regulation *in vivo* merits further investigation.

Is it possible that structural aberrations are not random, preferentially occurring at positions of special mtDNA organization? Indeed, the 4,977 bp deletion has previously been shown to be flanked by simple repeat sequences with the tendency to form non-B DNA structures (Hou and Wei, 1998). Interestingly, non-B DNA structures tend to co-localize in general with other types of mtDNA deletions that accumulated with aging (Hou and Wei, 1996; Damas et al., 2012). Specifically, as already discussed above, G-quadruplex forming sequences tend to occur at such breakpoints (Dong et al., 2014), and affect mtDNA transcription *in vitro* (Hillen et al., 2017). Hence, it is logical to suggest the existence of mtDNA hotspots for aberrations. In the nuclear genome hot spots for chromosomal aberrations tend to occur in regions with special chromatin organization (Mishmar et al., 1999; Fungtammasan et al., 2012), which calls for assessing such connection in the mtDNA as well.

### Structural Differences in mtDNA Across Evolution

mtDNA aberrations do not only associate with human pathologies, but also led to changes in mtDNA gene order and content during the course of evolution. As an example, although the mitochondrial genome remained circular in most studied metazoans, it is linear in *Medusozoa* (Kayal et al., 2012). Secondly, although most vertebrate mtDNAs contain a non-coding region, which harbors most known regulatory elements, the chordate amphioxus nearly lacks a non-coding region (Spruyt et al., 1998; Boore et al., 1999), which prevents identification of the positions of orthologous regulatory elements. Third, fragmentation of the mtDNA into several co-segregating parts that together comprise the full gene content seen in vertebrates has been described in organisms such as lice (Shao et al., 2012) and certain nematodes (Phillips et al., 2016). Do such mtDNA rearrangements affect mtDNA regulation? A recent study of *in vivo* mtDNA transcription using the precision global run-on transcription-sequencing (PRO-seq) revealed, that although the mtDNA gene contents in *Drosophila* and *Caenorhabditis elegans* are nearly identical to that of humans, the gene order and gene content per mtDNA strand profoundly changed (Blumberg et al., 2017). We recently showed that such changes were accompanied by the emergence of a very different mtDNA transcriptional initiation and termination schemes *in vivo* (Blumberg et al., 2017). Specifically, we observed that in contrast to human mtDNA which harbors two heavy strand and one light strand transcriptional initiation sites, Drosophila had 5-7 initiation sites, and C elegans had a single transcription initiation site, consistent with their mtDNA strand-gene contents. These phenomena exemplify how changes in mtDNA organization, during the course of evolution and in human diseases, likely lead to changes in mtDNA regulation.

As the recently identified mtDNA DNAse-seq and ATAC-seq footprinting patterns appears to be conserved between human and mouse (Blumberg et al., 2018), it would be of interest to study such in organisms with different mtDNA organization, as well as in human cells with pathological mtDNA deletions. Such study will directly assess the impact of mtDNA aberrations on mtDNA higher order organization and while engaging such study with techniques that assess transcriptional pattern *in vivo* (such as PRO-seq) one will be able to assess the connection between such changes with alteration in mtDNA regulation.

### The Management of mtDNA Structure—Mitochondrial Topoisomerases as Key Players

The structure of mtDNA and its accessibility is also impacted by topoisomerases, single or double-strand DNA-cleavage proteins that are used to alter the topological state of DNA, keeping it available for transcription and replication and preventing the formation of knots or other unusable structures (Vos et al., 2011). The issues faced by mtDNA that must be resolved by topoisomerase are distinct from those seen in linear chromosomes and include the resolution of concatameric structures formed by mtDNA replication (Kolesar et al., 2013).

There is a single known topoisomerase that is specific for the mitochondrion, TOP1MT (Zhang et al., 2001). This is a type IB topoisomerase, capable of relaxing supercoiling by single-strand cleavage and strand passage. Surprisingly, mice deleted for the homologous *Top1mt*, are viable, although they do show evidence of increased supercoiling of their mtDNA (Zhang et al., 2014). Instead, *Top1mt^−^^/^^−^* animals had increased activity of type IIA topoisomerases, suggesting the capacity for compensation for the loss of Top1mt activity.

The presence of type IIA topoisomerases is probably required in mitochondria, since these proteins fulfill the requirement for the de-catenation of linked molecules of mtDNA. Top2β has dual localization to the mitochondria and nucleus, with a shortened isoform present in the mitochondrion (Low et al., 2003). Top2β is canonically responsible for type IIA activity in non-proliferating cells. Although Top2α was not initially identified in the mitochondrion, recent studies have confirmed that it does locate within the organelle (Zhang et al., 2014).

The topoisomerases collectively appear to play important roles in regulating the supercoiling and also the transcription of mtDNA (Sobek et al., 2013). This provides a striking echo of our growing understanding of the role of topoisomerases in regulating nuclear transcription (McKinnon, 2016). The continuing studies, particularly of the bigenomic type IIA topoisomerases, may increase our understanding of how nuclear and mitochondrial transcription are coordinately regulated using template topology, a mechanism of control that is strikingly conserved from bacteria to man.

### MtDNA Methylation and Acetylation of TFAM

Nuclear chromatin is regulated by DNA and protein modifications including the methylation of cytosines and the acetylation of specific lysine residues in histones. Such changes directly correlate with chromatin accessibility and have antagonistic impact on gene expression: whereas H3K27 tri-methylation correlates with gene silencing, K27 acetylation correlates with gene activation (Rada-Iglesias et al., 2011). As histones are not imported into the mitochondria, there is considerable interest in the possibility that equivalent modifications occur in the mtDNA or proteins that bind to it. Recent work reported that acetylation and phosphorylation of TFAM can fine-tune TFAM-DNA binding affinity (King et al., 2018). However, as such results were obtained while testing the binding capacity of TFAM (modified and unmodified) to non-specific DNA, it still remains to be assessed whether such modifications affect TFAM binding to mtDNA in living cells. More intriguing is the discovery of several types of mtDNA methylation in different porcine tissues, which correlated with different patterns of mtDNA transcription and mtDNA copy numbers (Liu et al., 2019). The extent to which mtDNA CpG and GpC methylation affect mitochondrial function in cells and in the entire organism remains still in open discussion (Mposhi et al., 2017), and its very existence has been questioned (Matsuda et al., 2018). Nevertheless, there are reports of association between altered levels of mtDNA methylation and Alzheimer’s disease (Stoccoro et al., 2017), suggesting physiological relevance. Taken together, it seems that similar to the nuclear genome, the mtDNA might be ‘epigenetically’ modified, which correlates with downstream activity. However, the connection between such modifications and mitochondrial higher order organization, and with mitochondrial activities, still remains to be tested.

## Conclusions

The higher order organization of the bacterial nucleoid and the nuclear chromatin are tightly regulated, and the impact of such structures on regulation has been widely studied. In the current essay we discussed current knowledge of the higher order organization of the mitochondrial genome in light of evolution and of the growing usage of functional genomics techniques (**Figure 1**). Recent analysis of DNase-seq and ATAC-seq suggest a conserved mtDNA footprinting pattern between tissues, which does not correlate with the binding sites pattern of the only known mtDNA coating protein—TFAM. As such pattern is conserved between man and mouse, the time is ripe to hypothesize that mtDNA–protein interactions, and hence mtDNA higher order organization, are more complex, and more regulated, than once thought. As functional genomics techniques that determine interactions between genomic regions (such as HiC) grow gradually more sensitive, they could shed light on the packaging of this small genome, and its impact on regulation.

## Author Contributions

DM and NS conceived the idea and wrote the manuscript. RL and MN participated in critical reading. RL most contributed to writing the text discussing the bacterial nucleoid and nuclear chromatin. MN participated in writing the text discussing G-quadruplex.

## Funding

This study was funded by the Israeli Science Foundation grant 372/17, and by the US Army Life Sciences Division LS67993 grant, both awarded to DM.

## Conflict of Interest

The authors declare that the research was conducted in the absence of any commercial or financial relationships that could be construed as a potential conflict of interest.
